# The Female Athlete Triad/Relative Energy Deficiency in Sports (RED-S)

**DOI:** 10.1055/s-0041-1730289

**Published:** 2021-06-02

**Authors:** Alexandra Ruivo Coelho, Gonçalo Cardoso, Marta Espanhol Brito, Inês Neves Gomes, Maria João Cascais

**Affiliations:** 1 Maternidade Dr. Alfredo da Costa, Centro Hospitalar Universitário, Lisboa, Portugal; 2 Hospital Garcia de Orta, Lisboa, Portugal

**Keywords:** female athlete, low energy availability, amenorrhea, bone health, menstrual dysfunction, atleta feminina, baixa disponibilidade energética, amenorreia, saúde óssea, disfunção menstrual

## Abstract

In a healthy athlete, the caloric intake is sufficient for sports energy needs and body physiological functions, allowing a balance between energy availability, bone metabolism, and menstrual cycle. On the other hand, an imbalance caused by low energy availability due to a restrictive diet, eating disorders or long periods of energy expenditure leads to multisystemic deregulation favoring the essential functions of the body. This phenomenon, described as the female athlete triad, occurs in a considerable percentage of high-performance athletes, with harmful consequences for their future. The present review was carried out based on a critical analysis of the most recent publications available and aims to provide a global perception of the topic relative energy deficit in sport (RED-S). The objective is to promote the acquisition of more consolidated knowledge on an undervalued theme, enabling the acquisition of preventive strategies, early diagnosis and/or appropriate treatment.

## Resumo

Em uma atleta saudável, o aporte calórico é suficiente para a necessidade energética esportiva e para as funções fisiológicas corporais, permitindo um equilíbrio entre disponibilidade energética (DE), metabolismo ósseo e função menstrual. Por outro lado, um desequilíbrio devido à baixa disponibilidade energética (BDE) por dieta restritiva, perturbações alimentares ou grandes períodos de gasto energético conduz a uma desregulação multissistêmica priorizando as funções essenciais do corpo. Este fenômeno, descrito inicialmente como tríade da mulher atleta e, atualmente, como défice energético relativo no esporte (RED-S, na sigla em inglês) tem como pilares a BDE, disfunção menstrual e alterações na densidade mineral óssea (DMO), estando presente em uma percentagem considerável de atletas de alta competição, com consequências nefastas para o seu futuro a curto, médio e longo prazo. A presente revisão foi realizada a partir da análise crítica das publicações mais recentes disponíveis e pretende proporcionar uma percepção global do tema RED-S. O objetivo é promover a aquisição de um conhecimento mais consolidado sobre uma temática subvalorizada, possibilitando a aquisição de estratégias preventivas, diagnóstico precoce e/ou tratamento adequado.

## Definition: Female Athlete Triad versus Relative Energy Deficiency in Sport

The female athlete triad, initially described in 1993 and conceptually defined in 1997 by the American College of Sports Medicine (ACSM), was based on the presence of eating disorders, amenorrhea and osteoporosis. In 2007, 3 new components were defined: low energy availability (LEA), menstrual dysfunction, and changes in bone mineral density. 1 2 3 Afterwards, it was concluded that the existence of all elements for its diagnosis was not essential, since there is a very high variety in incidence for each one and that it is dependent on the type of sport, which can lead to underdiagnoses. Therefore, since 2014, after meeting the International Olympic Committee (IOC), it was changed for relative energy deficiency in sport (RED-S,) meeting the need for a holistic approach. 4 5

This new concept allows the identification of energy deficiency as a key to the disruption of several physiological functions of different areas, such as reproduction, bone, endocrine, metabolic, hematological, growth and development, physiological, cardiovascular, gastrointestinal, and immunological, with consequences for the performance and health of the athlete in general. 4 6

## Low Energy Availability

Low energy availability, due to food scarcity or excessive energy expenditure, causes physiological adaptations to ensure life maintenance. 7 Therefore, there are different mechanisms favoring essential processes 8 instead of secondary functions such as growth, development, and reproduction. 9 Energy availability (EA) is calculated by subtracting the energy consumed (kcal) from the energy ingested (kcal) and dividing this value by the free fat mass (kg). 10 It consists of a theoretical concept, difficult to use on a routine basis. However, its knowledge and interpretation are important for a better evaluation of the athletes. The ideal EA should support the basic functions that allow a healthy state and adequate performance, 4 which is believed to be > 45kcal/kg of free fat mass/day. 7 Several authors have attempted to define the threshold beyond which LEA leads to metabolic changes. However, due to the high interpersonal variability, it is only possible to predict that < 30kcal/kg of free fat mass/day, there is a high probability of physiological adaptation favoring vital systems. 4 7

## Pathophysiology

The new LEA concept highlights the complexity of this theme, which involves several hormonal pathways. There has been extensive research in this area in an attempt to identify the trigger of pathophysiological changes. However, these appear to result from multiple changes with different influences on different organs and systems.

## Adaptation to Energy Restriction

Low energy availability leads to a decrease in body fat mass with adaptation of normal adipose tissue activity and activation of different pathways after recognition as an internal stress state (namely activation of the hypothalamic-pituitary-adrenal [HPA] axis and the autonomic nervous system). These changes lead to neuroendocrine adaptations with energy redistribution in favor of vital systems preservation. 11 12 We can, therefore, identify:

- Decrease leptin 13 14 : anorexigenic hormone secreted by adipocytes and regulated by energy state. Negative impact on gonadotropin-releasing hormone (GnRH) secretion. 15
- Increased ghrelin 13 16 17 : orexigenic hormone secreted by gastric oxyntic cells. Levels inversely related to fat mass. It has an effect on the hypothalamus and on the pituitary gland, negatively affecting the secretion of GnRH, of adrenocorticotrophic hormone (ACTH), of growth hormone (GH), of follicle stimulating hormone (FSH), and of luteinizing hormone (LH).
- Increased peptide YY: increased resistance to ghrelin. Associated with decreased release of GnRH and gonadotropins. 18
- Decreased oxytocin 19 : apparently suppressive role in activity of the HPA axis and modifies the glucoregulatory response to caloric consumption. 20 It has antidepressant and anxiolytic effects. 21
- Decreased insulin with increased sensitivity. 22 its decrease has an negative influence on GnRH signaling.
- Decreased insulin-like growth factor 1 (IGF-I): stimulates osteoblast function and bone formation. It mediates several actions by GH and may be responsible for increasing resistance to it. 23 24 25 26 27
- Resistance to GH, despite its increase 23 24 : pituitary peptide, necessary for muscle and bone anabolism and metabolism of carbohydrates, proteins, and lipids.
- Thyroid function: adaptation due to decreased energy expenditure with a decrease in T3 and thyrotropin-releasing hormone (TRH). Thyroxine and thyroid-stimulating hormone (TSH) without changes/lower limit of normal. 8 28 29 30 31
- Activation of HPA axis: An increase in basal cortisol leads to an increase in its nocturnal pulse amplitude, half-life, and area below the curve in amenorrhea athletes. 32 33 Increase in beta-hydroxybutyrate (ketone synthesized in the liver: carrier of energy to peripheral tissues, activity as an energetic metabolite, cellular signaling functions). Cellular function, regardless of sports practice. 6

## Hypothalamic Amenorrhea

**Figs. 1 and 2.**
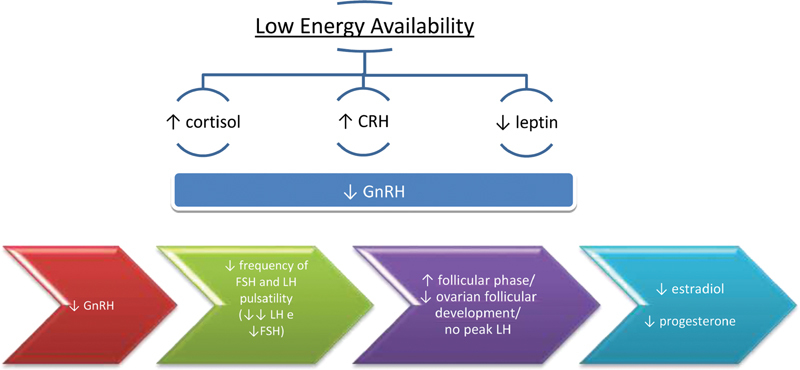
Influence of low energy availability on the menstrual cycle.

As previously explained, exercise by its own has no suppressive effect on reproductive function. However, it can be the cause of menstrual disruptions by influencing energy availability. According to different studies, it is believed that functional hypothalamic amenorrhea occurs by the combination of different pathways in response to LEA, with a negative influence on GnRH: increased cortisol 34 and corticotropin-releasing hormone (CRH) 35 in response to stress and decreased leptin, with impact directly GnRH ( Fig. 1 ). Therefore, there is a decrease in the GnRH drive with reduction in frequency of FSH and LH pulsatility, 22 30 36 leading to changes in folliculogenesis and ovulatory function, resulting in lower estradiol and progesterone levels ( Fig. 2 ). 37

It is important to emphasize that there is a wide spectrum of possible menstrual patterns, namely ovulatory eumenorrhea, subclinical menstrual dysfunctions, and amenorrhea. 34 A higher rate of amenorrhea occurs in sports whose lean phenotype is imposed (gymnastics, running, among others). The prevalence of hypothalamic amenorrhea can be as high as 69%, compared with 2 to 5% in the general population. 37 38 39

## Bone Metabolism

Bone development is negatively affected by LEA, with a decrease of different elements such as estrogens (inhibition of osteoclasts and growth of osteoblasts), IGF-1 (stimulation of osteoblastogenesis and promotion of bone formation), leptin (proliferation of osteoblasts) and T3 (proliferation of osteoblasts and promoting bone formation). Decrease in bone formation and bone turnover are the main consequences of changes in bone metabolism. This combination leads to loss of normal repair mechanisms for minor and major lesions, resulting in a higher risk of fracture. 40 41 42 43 In amenorrhea athletes, there is a decrease in bone mineral density (BMD), volumetric bone density, and strength associated with abnormal bone microarchitecture. 44 45 46 47 Even in weight-bearing exercises with theoretical benefit in BMD, changes are described mainly when associated with restrictive eating habits and low weight. 48 49

## Other Consequences

Cardiovascular : increase in total cholesterol, triglycerides, LDL and HDL. 50 Impairment of endothelial function and increased vascular resistance 51 52 53 associated with increased central fat. 54 Amenorrheic athletes can have lower heart rates and systolic blood pressure, due to disruptions of the normal renin-angiotensin-aldosterone response. 4 In more severe LEA states, severe bradycardia, hypotension, valve abnormalities, pericardial effusion, and arrhythmias can occur. 4

Sports performance : impaired recovery with change in muscle mass and function 55 ; interference in the glycogen reserve and protein synthesis. 56 The literature in this area is scarce, with only one study confirming a 10% decrease in the swimming speed of 400 m in athletes with amenorrhea versus eumenorrhea. 57

Bone metabolism : Because it occurs mostly in adolescence, there is a proven risk of loss of bone mass with potential inability to reach the bone peak, which in 90% of individuals is reached by age of 18. 6

## Diagnosis

The diagnosis of RED-S does not imply the existence of concrete clinical or laboratory changes. It consists of an active search for athletes at risk due to insufficient energy availability, either due to low input or excessive expenditure.

The diagnosis requires a low threshold of suspicion and an approach based on a detailed medical history that should include questions about diet, dietary changes, weight fluctuations, exercise, training hours, sleep changes, stress, mood, cycle menstruation, fractures, and substance abuse. It is also important to address psychosocial issues such as the need for social approval, the claim to perfectionism, ambitions and expectations, which are more marked in athletes with amenorrhea. Family history of eating and reproductive disorders should also be explored. 34 In order to standardize screening and follow-up, the IOC created the RED-S clinical assessment tool (CAT), which should be part of the annual health assessment of the athlete and/or whenever there is evidence of eating disorder, menstrual dysfunction (secondary amenorrhea >  6 months or primary amenorrhea > 16 years), history of stress fracture, significant weight loss, change in height in relation to the target family height in adolescents, deficient performance, or evident mood change. 6 58

Frequently, the first manifestation results in dysregulation of the menstrual cycle or in amenorrhea, and a functional etiology must be considered in the presence of oligomenorrhea and/or in the presence of amenorrhea for > 3 months. 6 34

Functional hypothalamic amenorrhea is characterized by the absence of menstrual cycles or by irregular cycles associated with estrogen deficiency due to insufficient stimulation or suppression of the hypothalamic-pituitary-ovary (HPO) axis in the absence of anatomical or organic pathology. 34

As it is an exclusion diagnosis, it is crucial to consider the main causes of amenorrhea, such as drugs, intracranial prolactinoma/tumor, Kallmann Syndrome (anosmia and hyposmia), thyroid pathology, chronic pathology, and congenital pathology (i.e., imperforate hymen, Mullerian abnormalities/androgen insensitivity syndrome [AIS]). 59 60 61 62 63 To complement this study, a complete physical examination with search for excess androgens signs is essential, and the need for gynecological evaluation and imaging studies should be considered. 29 64 65 66

In hypothalamic functional amenorrhea, bradycardia, orthostatic hypotension, BMI < 18.5 kg / m2, parotid hypertrophy and/or signs of tissue hypoperfusion 65 are frequently accompanied by signs of hypoestrogenism, namely delayed puberty, breast atrophy, and vaginal atrophy. 6

## Complementary Diagnostic Tests

The initial study of amenorrhea includes the evaluation of hCG levels (to exclude pregnancy), FSH, prolactin and TSH. Some authors also recommend in the initial assessment the search of Free T4 (FT4), LH, estradiol and anti-Müllerian hormone (AMH). 34

Additional studies depend on clinical suspicion. In chronic disease or eating disorder: blood count, liver and kidney function, electrolyte Panel, calcium, magnesium, phosphorus, glycemia, erythrocyte sedimentation rate, and C-reactive protein should be requested 34 ; in the presence of signs of hyperandrogenism, total testosterone, DHEA-S, and 17OH-progesterone should be considered. 67 68

Expected results in functional amenorrhea are decreased LH or low normal, normal FSH (usually higher than LH) and E2 < 50pg/ml. The acute response after GnRH stimulation is preserved. Thyroid stimulating hormone, FT4, and testosterone are usually at the lower limit of normal. 30 31 34 69 Anti-Müllerian hormone does not change, and there seems to be no interference in the ovarian reserve. 70

Bone mineral density assessment should be considered when menstrual dysfunction (6–12 months of amenorrhea/oligomenorrhea, primary amenorrhea), low BMI (< 17.5 kg/m2) or significant weight loss (> 5–10% of body mass within 1 month), in the presence of minor stress/post-trauma fracture, or in the event of an eating disorder. It is advisable to use the Z-Score scale in preference to T-Score, as the first is adapted to age and gender. Any location in the skeleton can be assessed; however, the spine is the most consensual in adolescents and young women with amenorrhea. 6 44 45 71 72 73 74

The interpretation of densitometry in athletes must follow specific criteria, with BMD being considered lower than expected when Z-score> -1; low BMD if Z-score between -1 and -1.9 with risk factors (nutritional deficiencies, hypoestrogenism or stress fractures), and osteoporosis when Z-score < -2 with risk factors. 2 3 49

## Treatment

Due to the multifactorial etiology of female athlete triad - stress, weight loss, excessive exercise, and poor nutrition - a multidisciplinary team is essential for its approach. 2 3

## Nonpharmacological

Nonpharmacological treatment is always the first line of treatment, allowing resolution of most cases. As it is based on LEA, the aim is to restore normal balance with an individualized and dynamic nutritional, psychological, and sports plan that will allow the reestablishment of the hypothalamic-pituitary-ovary axis. 4 The increase of 5 to 10% of body weight or of 1 to 4 kg of weight with appropriate nutritional supply – 300 to 600 kcal caloric increase – distributed throughout the day and with protein and carbohydrate consumption preference. 6

## Vitamin Supplementation

Calcium and vitamin D have shown important benefits in decreasing the risk of stress fractures, as well as in their recovery, with supplementation recommended. 75 76 A daily dose of 1,300 mg of calcium (up to ,1500 mg/day) and of 800-1,000 IU of vitamin D (to achieve blood 25[OH]D concentration > 75-100 nmol/L) 2 77 78 is recommended. The use of bisphosphonates should still be avoided, especially in young athlete women, considering its long half-life and its potential teratogenic effect in future pregnancies.

## Pharmacological

Pharmacological treatment has a crucial role in selected cases. However, it should only be considered after failure in reestablishing menstruation after between 6 and 12 months of nonpharmacological therapy associated with a proven decrease in BMD. In presence of a young athlete with amenorrhea or oligomenorrhea, combined oral contraception is often used as an adjunct to normalize menstrual cycles. Despite the success in most cases, its use for this purpose is not recommended, as it may cover a possible physiological normalization and give false confidence to the athlete. 4 Corroborating the nonindication for its prescription, several studies report the lack of efficacy of oral estrogens in the recovery of BMD/bone protective effect. 79 80 This is justified by their hepatic “first-pass effect,” with potential suppression of liver production of IGF-1 impairing its bone trophic effect. 6 81 Currently, when it is necessary to initiate hormone replacement, the most accepted approach consists of transdermal estradiol therapy (E2) (which does not affect IGF-I secretion) associated with a cyclic oral progestative for a short period. 34 Nevertheless, it is always important to remind athletes that this has no contraceptive effect. 82 For contraceptive purposes, there is no contraindication for any method, although if there is a preference for combined contraceptive, we can offer the vaginal or transdermal route to avoid hepatic “first-pass effect”. The only method that allows the perception of normal recovery are nonhormonal methods, such as nonhormonal intrauterine devices.

## Investigational Therapy

Recombinant parathyroid hormone : can be weighted for short periods of time when BMD is very low or in cases of delayed fracture healing. 34 It is contraindicated in adolescents or young adults with open growth plates. It has shown improvement in BMD and faster recovery. 83 84

Recombinant leptin : a promising therapy showing increased frequency and levels of LH pulse, improved follicular development, ovarian volume, E2 levels, increased T4, FT4, IGF-I, IGF-binding protein 3, bone alkaline phosphatase, and osteocalcin. However, decreased appetite and significant weight loss have been reported. 85 Similar results can be achieved with metreleptin regarding weight loss and fat mass. 86

## Recovery after Treatment

Nonpharmacological therapy can restore menstrual cycles to normal in months. However, some athletes may maintain folliculogenesis and altered follicular dynamics for years, with decreased gonadotropins and sex steroid hormones. In these cases, a luteal phase defect may occur with long menstrual periods associated with premenstrual spotting or short cycles due to decreased progesterone secretion. 87 88 Regarding bone metabolism, results can take several years to appear. Regardless of the positive correlation between increasing BMD and menstrual reestablishment, in many cases a full recovery is not achieved. 89

## Conclusion

Relative energy deficiency in sport consists of a low energy availability status mainly affecting young athletes, with potentially harmful and irreversible consequences on their health. Its prevalence is underestimated due to lack of and late diagnosis due to deficient knowledge of signs and symptoms. It is crucial and urgent to promote dissemination among different professionals, extending to athletes and their families, in order to increase alertness to this condition, allowing its prevention, early diagnosis, and adequate treatment.

## References

[1] Yeager K K Agostini R Nattiv A Drinkwater B The female athlete triad: disordered eating, amenorrhea, osteoporosis Med Sci Sports Exerc 1993 25 07 775 777 10.1249/00005768-199307000-00003 8350697

[2] American College of Sports Medicine Nattiv A Loucks A B Manore M M Sanborn C F Sundgot-Borgen J Warren M P American College of Sports Medicine position stand. The female athlete triad Med Sci Sports Exerc 2007 39 10 1867 1882 10.1249/mss.0b013e318149f111 17909417

[3] Expert Panel De Souza M J Nattiv A Joy E Misra M Williams N I Mallinson R J 2014 Female Athlete Triad Coalition Consensus Statement on Treatment and Return to Play of the Female Athlete Triad: 1st International Conference held in San Francisco, California, May 2012 and 2nd International Conference held in Indianapolis, Indiana, May 2013 Br J Sports Med 2014 48 04 289 10.1136/bjsports-2013-093218 24463911

[4] Mountjoy M Sundgot-Borgen J K Burke L M Ackerman K E Blauet C Constantini N IOC consensus statement on relative energy deficiency in sport (RED-S): 2018 update Br J Sports Med 2018 52 11 687 697 10.1136/bjsports-2018-099193 29773536

[5] Mountjoy M Sundgot-Borgen J Burke L Carter S Constantini N Lebrun C The IOC consensus statement: beyond the Female Athlete Triad--Relative Energy Deficiency in Sport (RED-S) Br J Sports Med 2014 48 07 491 497 10.1136/bjsports-2014-093502 24620037

[6] Lages A S Rebelo-Marques A R Carrilho F Défice Energético Relativo no Desporto (RED-S) Rev Med Desportiva Inf 2018 9 05 14 16 10.23911/Defice_Energetico_Relativo_no_Desporto

[7] Loucks A B Thuma J R Luteinizing hormone pulsatility is disrupted at a threshold of energy availability in regularly menstruating women J Clin Endocrinol Metab 2003 88 01 297 311 10.1210/jc.2002-020369 12519869

[8] Pauli S A Berga S L Athletic amenorrhea: energy deficit or psychogenic challenge? Ann N Y Acad Sci 2010 1205 33 38 10.1111/j.1749-6632.2010.05663.x 20840250 PMC2941235

[9] Dufour D L Sauther M L Comparative and evolutionary dimensions of the energetics of human pregnancy and lactation Am J Hum Biol 2002 14 05 584 602 10.1002/ajhb.10071 12203813

[10] Loucks A B Low energy availability in the marathon and other endurance sports Sports Med 2007 37(4-5):348 352 10.2165/00007256-200737040-00019 17465605

[11] Rodriguez-Pacheco F Martinez-Fuentes A J Tovar S Pinilla L Tena-Sempere M Dieguez C Regulation of pituitary cell function by adiponectin Endocrinology 2007 148 01 401 410 10.1210/en.2006-1019 17038552

[12] Mitchell M Armstrong D T Robker R L Norman R J Adipokines: implications for female fertility and obesity Reproduction 2005 130 05 583 597 10.1530/rep.1.00521 16264089

[13] Ackerman K E Slusarz K Guereca G Pierce L Slattery M Mendes N Higher ghrelin and lower leptin secretion are associated with lower LH secretion in young amenorrheic athletes compared with eumenorrheic athletes and controls Am J Physiol Endocrinol Metab 2012 302 07 E800 E806 10.1152/ajpendo.00598.2011 22252944 PMC3330709

[14] Franks P W Farooqi I S Luan J Wong M-Y Halsall I O'Rahilly S Does physical activity energy expenditure explain the between-individual variation in plasma leptin concentrations after adjusting for differences in body composition? J Clin Endocrinol Metab 2003 88 07 3258 3263 10.1210/jc.2002-021426 12843173

[15] Donato J Jr Cravo R M Frazão R Gautron L Scott M M Lachey J Leptin's effect on puberty in mice is relayed by the ventral premammillary nucleus and does not require signaling in Kiss1 neurons J Clin Invest 2011 121 01 355 368 10.1172/JCI45106 21183787 PMC3007164

[16] Misra M Miller K K Kuo K Griffin K Stewart V Hunter E Secretory dynamics of ghrelin in adolescent girls with anorexia nervosa and healthy adolescents Am J Physiol Endocrinol Metab 2005 289 02 E347 E356 10.1152/ajpendo.00615.2004 15755766

[17] Christo K Cord J Mendes N Miller K K Goldstein M A Klibanski A Acylated ghrelin and leptin in adolescent athletes with amenorrhea, eumenorrheic athletes and controls: a cross-sectional study Clin Endocrinol (Oxf) 2008 69 04 628 633 10.1111/j.1365-2265.2008.03237.x 18331605 PMC3206259

[18] Scheid J L De Souza M J Menstrual irregularities and energy deficiency in physically active women: the role of ghrelin, PYY and adipocytokines Med Sport Sci 2010 55 82 102 10.1159/000321974 20956862

[19] Lawson E A Ackerman K E Estella N M Estella N M Guereca G Pierce L Nocturnal oxytocin secretion is lower in amenorrheic athletes than nonathletes and associated with bone microarchitecture and finite element analysis parameters Eur J Endocrinol 2013 168 03 457 464 10.1530/EJE-12-0869 23258269 PMC3679669

[20] Lawson E A The effects of oxytocin on eating behaviour and metabolism in humans Nat Rev Endocrinol 2017 13 12 700 709 10.1038/nrendo.2017.115 28960210 PMC5868755

[21] Afinogenova Y Schmelkin C Plessow F Thomas J J Pulumo R Micali N Low fasting oxytocin levels are associated with psychopathology in anorexia nervosa in partial recovery J Clin Psychiatry 2016 77 11 e1483 e1490 10.4088/JCP.15m10217 28076675 PMC6124659

[22] Rickenlund A Thorén M Carlström K von Schoultz B Hirschberg A L Diurnal profiles of testosterone and pituitary hormones suggest different mechanisms for menstrual disturbances in endurance athletes J Clin Endocrinol Metab 2004 89 02 702 707 10.1210/jc.2003-030306 14764784

[23] Misra M Klibanski A Endocrine consequences of anorexia nervosa Lancet Diabetes Endocrinol 2014 2 07 581 592 10.1016/S2213-8587(13)70180-3 24731664 PMC4133106

[24] Laughlin G A Yen S S Nutritional and endocrine-metabolic aberrations in amenorrheic athletes J Clin Endocrinol Metab 1996 81 12 4301 4309 10.1210/jcem.81.12.8954031 8954031

[25] Gordon C M Goodman E Emans S J Grace E Becker K A Rosen C J Physiologic regulators of bone turnover in young women with anorexia nervosa J Pediatr 2002 141 01 64 70 10.1067/mpd.2002.125003 12091853

[26] Trombetti A Richert L Herrmann F R Chevalley T Graf J D Rizzoli R Selective determinants of low bone mineral mass in adult women with anorexia nervosa Int J Endocrinol 2013 2013 897193 10.1155/2013/897193 23634145 PMC3619547

[27] Misra M Miller K K Bjornson J Hackman A Aggarwal A Chung J Alterations in growth hormone secretory dynamics in adolescent girls with anorexia nervosa and effects on bone metabolism J Clin Endocrinol Metab 2003 88 12 5615 5623 10.1210/jc.2003-030532 14671143

[28] Estour B Germain N Diconne E Frere D Cottet-Emard J-M Carrot G Hormonal profile heterogeneity and short-term physical risk in restrictive anorexia nervosa J Clin Endocrinol Metab 2010 95 05 2203 2210 10.1210/jc.2009-2608 20305007

[29] Gordon C M Clinical practice. Functional hypothalamic amenorrhea N Engl J Med 2010 363 04 365 371 10.1056/NEJMcp0912024 20660404

[30] Berga S L Mortola J F Girton L Suh B Laughlin G Pham P Neuroendocrine aberrations in women with functional hypothalamic amenorrhea J Clin Endocrinol Metab 1989 68 02 301 308 10.1210/jcem-68-2-301 2493024

[31] Michopoulos V Mancini F Loucks T L Berga S L Neuroendocrine recovery initiated by cognitive behavioral therapy in women with functional hypothalamic amenorrhea: a randomized, controlled trial Fertil Steril 2013 99 07 2084 910 10.1016/j.fertnstert.2013.02.036 23507474 PMC3672390

[32] Schorr M Lawson E A Dichtel L E Klibanski A Miller K K Cortisol measures across the weight spectrum J Clin Endocrinol Metab 2015 100 09 3313 3321 10.1210/JC.2015-2078 26171799 PMC4570173

[33] Ackerman K E Patel K T Guereca G Pierce L Herzog D B Misra M Cortisol secretory parameters in young exercisers in relation to LH secretion and bone parameters Clin Endocrinol (Oxf) 2013 78 01 114 119 10.1111/j.1365-2265.2012.04458.x 22671919 PMC3443505

[34] Gordon C M Ackerman K E Berga S L Kaplan J R Mastorakos G Misra M Functional hypothalamic amenorrhea: an endocrine society clinical practice guideline J Clin Endocrinol Metab 2017 102 05 1413 1439 10.1210/jc.2017-00131 28368518

[35] Martin B Golden E Carlson O D Egan J M Mattson M P Maudsley S Caloric restriction: impact upon pituitary function and reproduction Ageing Res Rev 2008 7 03 209 224 10.1016/j.arr.2008.01.002 18329344 PMC2634963

[36] Elliott-Sale K J Tenforde A S Parziale A L Holtzman B Ackerman K E Endocrine effects of relative energy deficiency in sport Int J Sport Nutr Exerc Metab 2018 28 04 335 349 10.1123/ijsnem.2018-0127 30008240

[37] COUNCIL ON SPORTS MEDICINE AND FITNESS Weiss Kelly A K Hecht S The female athlete triad Pediatrics 2016 138 02 e20160922 10.1542/peds.2016-0922 27432852

[38] Nichols J F Rauh M J Barrack M T Barkai H S Pernick Y Disordered eating and menstrual irregularity in high school athletes in lean-build and nonlean-build sports Int J Sport Nutr Exerc Metab 2007 17 04 364 377 10.1123/ijsnem.17.4.364 17962711

[39] Beals K A Hill A K The prevalence of disordered eating, menstrual dysfunction, and low bone mineral density among US collegiate athletes Int J Sport Nutr Exerc Metab 2006 16 01 1 23 10.1123/ijsnem.16.1.1 16676700

[40] Hotta M Fukuda I Sato K Hizuka N Shibasaki T Takano K The relationship between bone turnover and body weight, serum insulin-like growth factor (IGF) I, and serum IGF-binding protein levels in patients with anorexia nervosa J Clin Endocrinol Metab 2000 85 01 200 206 10.1210/jcem.85.1.6321 10634387

[41] Dominguez J Goodman L Sen Gupta S Mayer L Etu S F Walsh B T Treatment of anorexia nervosa is associated with increases in bone mineral density, and recovery is a biphasic process involving both nutrition and return of menses Am J Clin Nutr 2007 86 01 92 99 10.1093/ajcn/86.1.92 17616767

[42] Viapiana O Gatti D Dalle Grave R Todesco T Rossini M Braga V Marked increases in bone mineral density and biochemical markers of bone turnover in patients with anorexia nervosa gaining weight Bone 2007 40 04 1073 1077 10.1016/j.bone.2006.11.015 17240212

[43] Ihle R Loucks A B Dose-response relationships between energy availability and bone turnover in young exercising women J Bone Miner Res 2004 19 08 1231 1240 10.1359/JBMR.040410 15231009

[44] Ackerman K E Nazem T Chapko D Russell M Mendes N Taylor A P Bone microarchitecture is impaired in adolescent amenorrheic athletes compared with eumenorrheic athletes and nonathletic controls J Clin Endocrinol Metab 2011 96 10 3123 3133 10.1210/jc.2011-1614 21816790 PMC3200253

[45] Mitchell D M Tuck P Ackerman K E Sokoloff N C Woolley R Slattery M Altered trabecular bone morphology in adolescent and young adult athletes with menstrual dysfunction Bone 2015 81 24 30 10.1016/j.bone.2015.06.021 26123592 PMC4745258

[46] Ackerman K E Putman M Guereca G Taylor A P Pierce L Herzog D Cortical microstructure and estimated bone strength in young amenorrheic athletes, eumenorrheic athletes and non-athletes Bone 2012 51 04 680 687 10.1016/j.bone.2012.07.019 22878154 PMC3482939

[47] Ackerman K E Cano Sokoloff N DE Nardo Maffazioli G Clarke H M Lee H Misra M Fractures in relation to menstrual status and bone parameters in young athletes Med Sci Sports Exerc 2015 47 08 1577 1586 10.1249/MSS.0000000000000574 25397605 PMC4430468

[48] Young N Formica C Szmukler G Seeman E Bone density at weight-bearing and nonweight-bearing sites in ballet dancers: the effects of exercise, hypogonadism, and body weight J Clin Endocrinol Metab 1994 78 02 449 454 10.1210/jcem.78.2.8106634 8106634

[49] Robinson T L Snow-Harter C Taaffe D R Gillis D Shaw J Marcus R Gymnasts exhibit higher bone mass than runners despite similar prevalence of amenorrhea and oligomenorrhea J Bone Miner Res 1995 10 01 26 35 10.1002/jbmr.5650100107 7747628

[50] Friday K E Drinkwater B L Bruemmer B Chesnut C III Chait A Elevated plasma low-density lipoprotein and high-density lipoprotein cholesterol levels in amenorrheic athletes: effects of endogenous hormone status and nutrient intake J Clin Endocrinol Metab 1993 77 06 1605 1609 10.1210/jcem.77.6.8263148 8263148

[51] O'Donnell E De Souza M J The cardiovascular effects of chronic hypoestrogenism in amenorrhoeic athletes: a critical review Sports Med 2004 34 09 601 627 10.2165/00007256-200434090-00004 15294009

[52] O'Donnell E Harvey P J Goodman J M De Souza M J Long-term estrogen deficiency lowers regional blood flow, resting systolic blood pressure, and heart rate in exercising premenopausal women Am J Physiol Endocrinol Metab 2007 292 05 E1401 E1409 10.1152/ajpendo.00547.2006 17227959

[53] Hoch A Z Papanek P Szabo A Widlansky M E Schimke J E Gutterman D D Association between the female athlete triad and endothelial dysfunction in dancers Clin J Sport Med 2011 21 02 119 125 10.1097/JSM.0b013e3182042a9a 21358502 PMC3570811

[54] Puder J J Monaco S E Sen Gupta S Wang J Ferin M Warren M P Estrogen and exercise may be related to body fat distribution and leptin in young women Fertil Steril 2006 86 03 694 699 10.1016/j.fertnstert.2006.02.085 16814292

[55] Fogelholm M Effects of bodyweight reduction on sports performance Sports Med 1994 18 04 249 267 10.2165/00007256-199418040-00004 7817064

[56] Areta J L Burke L M Camera D M West D W Crawshaw S Moore D R Reduced resting skeletal muscle protein synthesis is rescued by resistance exercise and protein ingestion following short-term energy deficit Am J Physiol Endocrinol Metab 2014 306 08 E989 E997 10.1152/ajpendo.00590.2013 24595305

[57] Vanheest J L Rodgers C D Mahoney C E De Souza M J Ovarian suppression impairs sport performance in junior elite female swimmers Med Sci Sports Exerc 2014 46 01 156 166 10.1249/MSS.0b013e3182a32b72 23846160

[58] Melin A Tornberg A B Skouby S Faber J Ritz C Sjödin A The LEAF questionnaire: a screening tool for the identification of female athletes at risk for the female athlete triad Br J Sports Med 2014 48 07 540 545 10.1136/bjsports-2013-093240 24563388

[59] Perkins R B Hall J E Martin K A Neuroendocrine abnormalities in hypothalamic amenorrhea: spectrum, stability, and response to neurotransmitter modulation J Clin Endocrinol Metab 1999 84 06 1905 1911 10.1210/jcem.84.6.5823 10372685

[60] Thangavelu K Geetanjali S Menstrual disturbance and galactorrhea in people taking conventional antipsychotic medications Exp Clin Psychopharmacol 2006 14 04 459 460 10.1037/1064-1297.14.4.459 17115873

[61] Illingworth P Amenorrhea, anovulation, and dysfunctional uterine bleeding Philadelphia Saunders/Elsevier 2010 2341 55

[62] Rebar R Evaluation of amenorrhea, anovulation, and abnormal bleeding South Dartmouth MDText.com, Inc. 2000 25905367

[63] Bulun S E Physiology and pathology of the female reproductive axis 13th ed. Philadelphia Elsevier 2016 590 664

[64] Golden N H Carlson J L The pathophysiology of amenorrhea in the adolescent Ann N Y Acad Sci 2008 1135 163 178 10.1196/annals.1429.014 18574222

[65] Frumar A M Meldrum D R Judd H L Hypercarotenemia in hypothalamic amenorrhea Fertil Steril 1979 32 03 261 264 488406

[66] Lizneva D Suturina L Walker W Brakta S Gavrilova-Jordan L Azziz R Criteria, prevalence, and phenotypes of polycystic ovary syndrome Fertil Steril 2016 106 01 6 15 10.1016/j.fertnstert.2016.05.003 27233760

[67] Rosner W Auchus R J Azziz R Sluss P M Raff H Position statement: Utility, limitations, and pitfalls in measuring testosterone: an Endocrine Society position statement J Clin Endocrinol Metab 2007 92 02 405 413 10.1210/jc.2006-1864 17090633

[68] Pinola P Piltonen T T Puurunen J Vanky E Sundström-Poromaa I Stener-Victorin E Androgen profile through life in women with polycystic ovary syndrome: a Nordic multicenter collaboration study J Clin Endocrinol Metab 2015 100 09 3400 3407 10.1210/jc.2015-2123 26192874

[69] Warren M P Holderness C C Lesobre V Tzen R Vossoughian F Brooks-Gunn J Hypothalamic amenorrhea and hidden nutritional insults J Soc Gynecol Investig 1994 1 01 84 88 10.1177/107155769400100117 9419753

[70] La Marca A Pati M Orvieto R Stabile G Carducci Artenisio A Volpe A Serum anti-müllerian hormone levels in women with secondary amenorrhea Fertil Steril 2006 85 05 1547 1549 10.1016/j.fertnstert.2005.10.057 16616745

[71] International Society for Clinical Densitometry Crabtree N J Arabi A Bachrach L K Fewtrell M Fulheihan GE-H Kescskemethy H H Dual-energy X-ray absorptiometry interpretation and reporting in children and adolescents: the revised 2013 ISCD Pediatric Official Positions J Clin Densitom 2014 17 02 225 242 10.1016/j.jocd.2014.01.003 24690232

[72] Bachrach L K Guido D Katzman D Litt I F Marcus R Decreased bone density in adolescent girls with anorexia nervosa Pediatrics 1990 86 03 440 447 2388792

[73] Soyka L A Misra M Frenchman A Miller K K Grinspoon S Schoenfeld D A Abnormal bone mineral accrual in adolescent girls with anorexia nervosa J Clin Endocrinol Metab 2002 87 09 4177 4185 10.1210/jc.2001-011889 12213868

[74] Grinspoon S Thomas E Pitts S Gross E Mickley D Miller K Prevalence and predictive factors for regional osteopenia in women with anorexia nervosa Ann Intern Med 2000 133 10 790 794 10.7326/0003-4819-133-10-200011210-00011 11085841 PMC3206091

[75] Moreira C A Bilezikian J P Stress fractures: concepts and therapeutics J Clin Endocrinol Metab 2017 102 02 525 534 10.1210/jc.2016-2720 27732325

[76] Kim B Y Kraus E Fredericson M Serum vitamin D levels are inversely associated with time lost to bone stress injury in a cohort of NCAA division I distance runners Clin J Sport Med 2016 26 02 e61

[77] NIH Consensus Development Panel on Osteoporosis Prevention, Diagnosis, and Therapy Osteoporosis prevention, diagnosis, and therapy JAMA 2001 285 06 785 795 10.1001/jama.285.6.785 11176917

[78] De Souza M J Williams N I Beyond hypoestrogenism in amenorrheic athletes: energy deficiency as a contributing factor for bone loss Curr Sports Med Rep 2005 4 01 38 44 10.1007/s11932-005-0029-1 15659278

[79] Warren M P Brooks-Gunn J Fox R P Holderness C C Hyle E P Hamilton W G Persistent osteopenia in ballet dancers with amenorrhea and delayed menarche despite hormone therapy: a longitudinal study Fertil Steril 2003 80 02 398 404 10.1016/s0015-0282(03)00660-5 12909505

[80] Cobb K L Bachrach L K Sowers M Nieves J Greendale G A Kent K K The effect of oral contraceptives on bone mass and stress fractures in female runners Med Sci Sports Exerc 2007 39 09 1464 1473 10.1249/mss.0b013e318074e532 17805075

[81] Southmayd E A De Souza M J A summary of the influence of exogenous estrogen administration across the lifespan on the GH/IGF-1 axis and implications for bone health Growth Horm IGF Res 2017 32 2 13 10.1016/j.ghir.2016.09.001 27693042

[82] Ackerman K E Singhal V Baskaran C Transdermal 17-beta-estradiol has a beneficial effect on bone parameters assessed using HRpQCT compared to oral ethinyl estradiol-progesterone combination pills in oligoamenorrheic athletes: a randomized controlled trial J Bone Miner Res 2017 32 01 S41

[83] Fazeli P K Wang I S Miller K K Herzog D B Misra M Lee H Teriparatide increases bone formation and bone mineral density in adult women with anorexia nervosa J Clin Endocrinol Metab 2014 99 04 1322 1329 10.1210/jc.2013-4105 24456286 PMC3973785

[84] Zhang D Potty A Vyas P Lane J The role of recombinant PTH in human fracture healing: a systematic review J Orthop Trauma 2014 28 01 57 62 10.1097/BOT.0b013e31828e13fe 23454854

[85] Welt C K Chan J L Bullen J Murphy R Smith P DePaoli A M Recombinant human leptin in women with hypothalamic amenorrhea N Engl J Med 2004 351 10 987 997 10.1056/NEJMoa040388 15342807

[86] Chou S H Chamberland J P Liu X Matarese G Gao C Stefanakis R Leptin is an effective treatment for hypothalamic amenorrhea Proc Natl Acad Sci U S A 2011 108 16 6585 6590 10.1073/pnas.1015674108 21464293 PMC3080974

[87] Santoro N Update in hyper- and hypogonadotropic amenorrhea J Clin Endocrinol Metab 2011 96 11 3281 3288 10.1210/jc.2011-1419 22058375

[88] Loucks A B Verdun M Heath E M Low energy availability, not stress of exercise, alters LH pulsatility in exercising women J Appl Physiol (1985) 1998 84 01 37 46 10.1152/jappl.1998.84.1.37 9451615

[89] Cialdella-Kam L Guebels C P Maddalozzo G F Manore M M Dietary intervention restored menses in female athletes with exercise-associated menstrual dysfunction with limited impact on bone and muscle health Nutrients 2014 6 08 3018 3039 10.3390/nu6083018 25090245 PMC4145292

